# Blood Pressure Control and Associated Factors among Hypertension Comorbid Type 2 Diabetic Patients in Southeast Ethiopia

**DOI:** 10.1155/2024/6668436

**Published:** 2024-04-16

**Authors:** Fikreab Desta, Selamawit Mengesha, Fanuel Belayneh, Demelash Woldeyohannes, Yohannes Tekalegn, Demisu Zenbaba, Biniyam Sahiledengle, Dejene Hailu

**Affiliations:** ^1^Public Health Department, Madda Walabu University, Goba Referral Hospital, Bale Goba, Ethiopia; ^2^School of Public Health, College of Medicine and Health Sciences, Hawassa University, Hawassa, Ethiopia; ^3^Department of Public Health, College of Medicine and Health Sciences, Wachemo University, Hossana, Ethiopia

## Abstract

**Background:**

Hypertension is the main contributor to the morbidity and mortality of patients with cardiovascular disease. Even though hypertension is very common in comorbid type 2 diabetic patients, it is frequently overlooked. This study aimed to assess blood pressure control and its associated factors among hypertension comorbid type 2 diabetic patients in Bale Zone public hospitals in Southeast Ethiopia.

**Methods and Materials:**

A hospital-based cross-sectional study design was conducted among hypertension comorbid type 2 diabetic patients. The data were collected using an interviewer-administered structured questionnaire and a review of the medical charts of patients. A simple random sampling technique was used to select the study participants. The bivariate and multivariate logistic regression analyses were performed to assess the association between blood pressure control and its associated factors. Independent variables that showed a *P* < 0.25 in the bivariate analysis was included in the multivariate analysis. Finally, variables with a *P* < 0.05 were declared statistically significant factors.

**Results:**

The total number of participants in the study was 378. The overall magnitude of uncontrolled hypertension among hypertension comorbid diabetic patients was found to be 82.5% (95% CI: 78.7%, 86.4%). Nonadherence to antihypertensive medication (AOR = 2.45, 95% CI: 1.11, 5.39, *P* = 0.027), duration of hypertension >10 years (AOR = 5.2, 95% CI: 1.27, 21.38, *P* = 0.022), participants who attended secondary education (AOR = 3.2, 95% CI: 1.18, 8.87, *P* = 0.023), and being obese (AOR = 4.1, 95% CI: 1.24, 13.49, *P* = 0.021) were significantly associated with uncontrolled hypertension.

**Conclusion:**

Uncontrolled hypertension was found to be high among hypertension comorbid type 2 diabetic patients. Patients' adherence to antihypertensive medication, physical activity, and alcohol abstinence should be maximized. Loss of weight is also crucial, as is the early detection and management of comorbidities.

## 1. Background

Hypertension is a major global health concern and a major preventable risk factor for cardiovascular events that affects over one billion people worldwide [1, 2]. Hypertension, in particular, is responsible for nearly 45% of heart disease deaths and 51% of stroke deaths [3]. On a global scale, nearly 40% of adults aged 25 and older were estimated to have hypertension [3]. The global prevalence of hypertension ranges from 4% to 78% [4]. Hypertension is still an important public health problem; it will increase to 1.56 billion globally by 2025 [5].

According to the 2014-JNC-8 hypertension guideline, uncontrolled hypertension is defined as blood pressure greater than or equal to 140/90 mmHg [1, 6]. Hence, the hypertension level at which the availability of therapy reduces morbidity and mortality associated with hypertension is demonstrated in people aged 18 and older [7].

More than 80% of deaths from hypertension and related cardiovascular diseases occur in low- and middle-income countries, and people with low socioeconomic status are more likely to suffer from the problem [5]. Hypertension can occur together with other cardiometabolic conditions, namely, diabetes, stroke, dyslipidemia, insulin resistance, glucose intolerance, and obesity [4, 6]. Between 20 and 60% of individuals with type 2 DM will have concomitant uncontrolled hypertension [8]. Africa had the highest prevalence of hypertension among the WHO regions, at 46% for all adults combined in 2019 [9]. Unfortunately, if hypertension is not controlled, it can lead to blindness, myocardial infarction, dementia, cardiac failure, and stroke [5, 10, 11]. It is predicted to be responsible for 7.5 million deaths or about 12.8% of all deaths in sub-Saharan Africa each year [12–14].

Hypertension has rapidly increased in prevalence, affecting a large number of people in sub-Saharan Africa [11]. In sub-Saharan Africa, the prevalence ranges from 25.4% to 41.1% in men and 27.2% to 38.7% in women [15]. Hyperinsulinemia, extracellular fluid volume expansion, and increased arterial stiffness have also been proposed as contributing factors for the development of hypertension in diabetic patients; diabetes roughly doubles the risk of cardiovascular disease, and concurrent hypertension nearly doubles that risk again [16].

Based on the evidence provided by clinical trials, guidelines recommend that patients who have been diagnosed with cardiovascular disease and its equivalents should reduce their blood pressure to <140/90 mmHg (so do hypertension comorbid type 2 diabetic patients) [17].

Chronic noncommunicable diseases (NCDs) are becoming more prevalent in Ethiopia, creating a double burden on the population. Population-based studies showed a high prevalence of NCDs in both rural and urban settings, including hypertension, other cardiovascular diseases, and diabetes [18, 19]. However, the Ethiopian health system is primarily focused on communicable disease prevention and control, with little emphasis on noncommunicable diseases (NCDs) such as hypertension and diabetes [20].

The reported prevalence of hypertension in various Ethiopian regions varied significantly [4, 21]. Uncontrolled hypertension ranges from 11.4 to 69.9% among hypertensive patients on treatment in Ethiopia [22–24]. This may increase if the patient has type 2 diabetes; other studies have revealed that in Ethiopia, the prevalence of uncontrolled hypertension among adults increased alarmingly from 9.3% in 2011 to 23.5% in 2015 [25–27]. Only about 28.4% of patients were taking an antihypertensive drug [27]. In Ethiopia, 97% of hypertensive patients do not receive the proper preventive care or treatment; only 2.8% of them receive treatment, and only 1.5% have their hypertension under control [28, 29]. There are currently only a few published articles in Ethiopia that assess uncontrolled blood pressure in hypertension comorbid type 2 diabetic patients. As a result, the study aimed to assess blood pressure control and its associated factors among hypertension comorbid type 2 diabetic patients in Southeast Ethiopia.

## 2. Methods and Materials

### 2.1. Study Area, Period, and Setting

A study was conducted in public hospitals in the Bale Zone of Southeast Ethiopia. It is approximately 403 kilometers from Addis Ababa, the country's capital. During the data collection period, there were three hospitals in the Bale Zone: Goba Referral Hospital and two general hospitals (Dellomena General Hospital and Robe General Hospital). Approximately 3,308 adult HIV/AIDS patients registered for ART follow-up in public hospitals between February 19 and April 30, 2020/2021. A hospital-based cross-sectional study design was conducted among hypertension comorbid type 2 diabetic patients from February 19 to April 30, 2020. Three public hospitals serving a catchment area of about 3 million people each provided care for more than 25,000 inpatients and 90,000 outpatients annually. The study included all adults with co-existing type 2 diabetes and adult hypertension who were 18 years of age or older, received antihypertensive medication for at least 6 months prior to the study period, and were being monitored.

### 2.2. Sample Size and Sampling Procedures

The sample size was calculated using a single population proportion formula, assuming a 95% confidence interval (CI), a prevalence of 56.5% for uncontrolled hypertension taken from a study conducted at Jimma University Medical Center [30], a 5% margin of error, a 1.5 design effect, and a 10% nonresponse rate. The final sample size was 405, and a simple random sampling technique was used to select the study participants from the chronic disease follow-up clinic registration book, which consists of lists of 700 hypertension comorbid type 2 diabetic patients in both randomly selected hospitals (Robe General Hospital and Goba Referral Hospital).

### 2.3. Data Collection, Procedure, and Quality Control

Data were collected using a structured questionnaire adapted from the WHO's step-wise approach to sociodemographic profile, behavioral and physical measurements, risk factors, and chronic disease surveillance [31]. Blood pressure (BP) was measured by using a digital mercury sphygmomanometer on the upper arm in a sitting position with back support after study participants rested for at least 5 minutes. A measurement was made after ensuring that respondents had rested from vigorous work or exercise, avoided smoking, and not consumed caffeine and alcohol for the last 30 minutes; otherwise, the BP measurement was postponed for 30 minutes. The respondent was told to sit relaxed without crossing legs and to stop talking. Two consecutive measurements were made in the left arm with the respondents in the same position, with an interval of at least 10 minutes. The average systolic and diastolic blood pressures were determined after taking the two measurements and recorded. Height and weight were measured with subjects standing with bare feet and wearing light clothes. The weight was measured using a well-calibrated digital weighing scale. The height was measured using a portable stadiometer, which was considered anthropometric with a simple triangular headboard. In taking the height of the respondent, the adult was made to stand straight with his or her shoe off and head erected such that the external auditory meatus and the lower border of the eye were in one horizontal plane (Frankfurt plane). Height was recorded to the nearest 0.5 cm and weight to the nearest 100 gm. Medication adherence of the study participants was assessed using the Morisky Medication Adherence Scale (MMAS-8) which has eight items and from this, individuals who scored six and greater than six were adherent to antihypertensive drug [32]. Physical activity level is classified into moderate and vigorous physical activity. An activity that involves walking briskly, bicycling, swimming for recreation, dancing, or mowing for at least 30 minutes for at least 5 days per week was considered as moderate physical activity, whereas an activity that involves running, fast swimming, fast cycling, or carrying/moving heavy loads greater than 20 kg for at least 10 minutes continuously was considered as vigorous physical activity [33]. WHO recommends a reduction to <2 g/day salt intake to help reduce blood pressure in adults with hypertension (strong recommendation). Hence, we have used teaspoons to categorize the level of salt intake [34].

The questionnaire was prepared in English and then translated into the local language (Afaan Oromo). Data were collected by four trained nurses who speak and write the local language; patients' medical charts and medical records were reviewed after all data collection tools were pretested before actual data collection time; and corrections were made. The data were checked for completeness and accuracy, cleared, and coded before being exported to SPSS for analysis.

### 2.4. Data Processing and Analysis

The collected data were entered into EpiData version 3.1 and exported to the Statistical Package for Social Sciences (SPSS) version 21.0 for analysis. Categorical and continuous data were analyzed using descriptive statistics such as frequency, percentages, median, mean, and standard deviation, which were calculated as study variables to describe the result of the study. The bivariable and multivariable binary logistic regression analyses were performed to assess the association between dependent and independent variables using the crude odds ratio (OR) and adjusted odds ratio (AOR). Confounders who were theoretically significant, regardless of their *P* value, and variables with a *P* value of less than 0.25 in a bivariable analysis were included in the multivariable model to control for confounders. Model fitness was checked using the Hosmer–Lemeshow test. Finally, the variables that showed *P* < 0.05 were considered statistically significant.

## 3. Results

Of the total sample, 378 (93.3%) individuals participated in the study. Of the 378 study participants, 211 (55.8%) were male. The median age of the respondents was 47 years, 171 (45.2%) were >50 years old, and 307 (81.2%) were married. Of the 378 study participants, 165 (43.7%) were Muslim religion followers and 210 (55.6%) were Oromo in ethnicity (Table 1).

### 3.1. Clinical Characteristics of Participants

The clinical characteristics of patients showed that 202 (53.4%) study participants had a family history of hypertension and 183 (48.4%) had a family history of diabetes. Of the study participants, 197 (52.1%) and 198 (52.4%) patients were living with hypertension and diabetes for less than five years, respectively. The mean and standard deviation (mean ± SD) for the duration of hypertension were 6.65 years ± 4.61 years with a range of 1–23 years (Table 2).

### 3.2. Behavioral and Dietary Characteristics of Study Participants

Of the 378 study participants, 122 (32.3%) were alcohol drinkers, 61 (16.1%) were cigarette smokers, and 59 (15.6%) were daily current smokers; 51 (13.5%) had eaten high-calorie food (burger, pizza, and kukis), and about 272 (72%) had eaten animal sources of fatty flesh meat, with regard to physical exercise; only 43 (11.4%) study participants reported that they had been involved in vigorous intensive physical activity, and 208 (55%) were using less than one teaspoon of salt per day. Almost all study participants practiced unhealthy diets or high-calorie foods (Table 3).

### 3.3. Antihypertensive and Hypoglycemic Medications

The overall utilization of antihypertensive drugs showed that 230 (60.85%) patients were on dual antihypertensive treatment. Most of the patients, about 113 (29.89%), were on a combination of enalapril and amlodipine, followed by hydrochlorothiazide and enalapril (73 (19.31%)). About 7.41% of patients were taking triple antihypertensive drugs (Table 4).

### 3.4. The Magnitude of Uncontrolled Blood Pressure among Hypertensive Diabetic Patients

**Figure 1:**
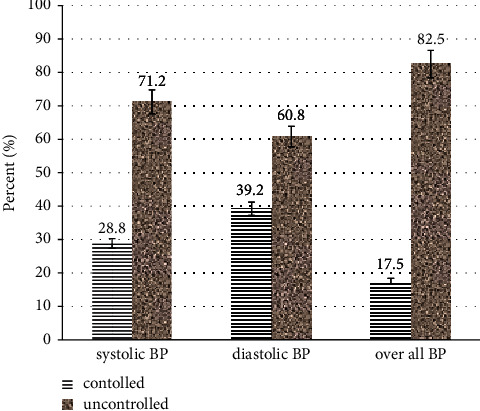
Magnitude of uncontrolled hypertension among hypertensive patients with diabetic comorbidity at public hospitals in Bale Zone, Southeast Ethiopia.

The mean and standard deviation (mean ± SD) of systolic blood pressure (SBP) were 142.35 ± 17.82, while the mean and standard deviation (mean ± SD) of diastolic blood pressure (DBP) were 87.58 ± 4.38. Of the 378 study participants, about 269 (71.2%) had an uncontrolled SBP, whereas about 39.2% had a controlled DBP. Overall uncontrolled hypertension was observed in 312 (82.5%) (95% CI: 78.7%, 86.4%) study participants (Figure 1).

### 3.5. Factors Associated with Uncontrolled Hypertension among Hypertensive Diabetic Patients

Factors associated with uncontrolled hypertension were examined using bivariable and multivariable binary logistic regression methods. In bivariable binary logistic regression analysis, age, educational level, occupation, duration with hypertension (HTN), duration with diabetes (DM), body mass index, comorbidities, and medication adherence risk factors showed associations with uncontrolled hypertension. Finally, the multivariable binary logistic regression technique was used for further analysis, and the level of education, body mass index, adherence level to antihypertensive drugs, and duration of hypertension with a duration greater than 10 years were significantly associated with uncontrolled hypertension (Table 5).

## 4. Discussion

According to our study, 82.5% of hypertensive diabetic patients had uncontrolled hypertension. Body mass index, duration of hypertension, and nonadherence to antihypertensive medication were all linked to uncontrolled hypertension.

In this study, the prevalence of hypertension was found to be 82.5%, which was higher than in previous studies at Jimma University Medical Center (56.49%) [30], Debre Tabor General Hospital (59.5%) [35], Morocco (70.4%) [36], a national diabetes center in Jordan (72.4%), Greece (55.6%) [37], and the USA (49.8%) [38]. This disparity in uncontrolled hypertension may be due to an increase in systolic and diastolic blood pressure with age. The majority of patients in this study were over the age of 50, which could have a negative impact on hypertension control as age increased [35, 39] and as patient being comorbid with type 2 diabets hinders hypertension control [30].

However, studies conducted in Canada at the Maritime Provinces among type 2 diabetic patients indicated the magnitude of uncontrolled hypertension were (78.7%) [40], in rural South Africa (75.5%) [41], Iraq (89.6%) [42], and Afro-Caribbean individuals living in the United Kingdom (82%) [43] had comparable levels of uncontrolled hypertension to those in the current study; this could be due to the presence of related comorbidities [16] as well as how patients adhere to their antihypertensive treatment plan.

According to the current study, patients who did not take their antihypertensive medication as prescribed were twice as likely to have uncontrolled hypertension as their counterparts (AOR 2.45, *P*=0.027). The finding is in line with the results obtained from studies from Jimma University Medical Center [30], South Africa [44], Ayder Comprehensive Specialized Hospital Tigray, Northern Ethiopia [45], the USA [38], and Nigeria [46]. This could have suggested that for hypertension comorbid type 2 diabetic patients, treatment compliance with antihypertensive medications is crucial to achieving target blood pressure levels [30]. Based on the findings of this study, patients were advised and encouraged to stick to their antihypertensive treatment, which is critical for achieving the recommended target blood pressure level (140/90 mmHg), especially in patients requiring intensive hypertension control, like comorbid diabetes.

This study found that patients with a history of hypertension for more than ten years were five times (AOR = 5.2, *P* = 0.022) more likely to have uncontrolled hypertension than those without a history of hypertension. This is supported by research from Jimma University Medical Center [30] and Tikur Anbessa General Specialized Hospital [23]; finding that diabetes and hypertension are common risk factors reported in other studies in other countries, the risk of hypertension increased as DM patients got older, which showed the risk of uncontrolled hypertension increased as diabetic patients ages increased [18].

In this study, body mass index (BMI) was also significantly associated with uncontrolled hypertension (AOR = 4.09). Participants in the study with a BMI of 30 kg/m^2^ were four times more likely to develop uncontrolled hypertension than those with a normal BMI. Similarly, obesity or being overweight increases the risk of developing hypertension. Several studies reveal that obesity is now emerging as a factor even in the poor population of developing countries [18, 45, 47].

### 4.1. Limitation of the Study

This study aimed to examine the determinants of uncontrolled hypertension in a study area with no prior evidence of the distribution and associated risk factors. Although the study used primary data on the magnitude of uncontrolled hypertension, medication adherence, and dietary assessments with trained data collectors and supervisors, the findings must be interpreted in light of the following limitations: first, due to the cross-sectional nature of the study, a cause-and-effect relationship cannot be established between the risk factors and uncontrolled hypertension; second, participants were assessed using self-reported surveys of adherence to drugs, cigarette smoking, and alcohol drinking, which might be liable to social desirability bias; and third, as it is hospital-based, the finding may not be generalizable to the total population. Due to the silent nature of the cases, future studies could use more objective measurements and qualitative studies to further explore factors related to behavioral aspects of hypertension and diabetes.

## 5. Conclusion

In this study, the magnitude of uncontrolled hypertension was found to be high among hypertension comorbid type 2 diabetic patients in Bale Zone public hospitals. The findings of this study call for local and national strategies, and more actions should be targeted for this group of patients to achieve target levels of blood pressure among hypertensive diabetic patients.

## Figures and Tables

**Table 1:** Sociodemographic characteristics of study participants at Bale Zone public hospitals, Southeast Ethiopia, 2020 (*n* = 378).

Variables	Frequency	Percentage
*Sex*		
Male	211	55.8
Female	167	44.2

*Age*		
≤30 years	21	5.6
31–40 years	100	26.5
41–50 years	86	22.7
>50 years	171	45.2

*Level of education*		
No formal education	133	35.2
Primary school	89	23.5
Secondary school	80	21.2
Tertiary and above	76	20.1

*Marital status*		
Single	31	8.2
Married	307	81.2
Divorced	7	1.9
Widowed	33	8.7

*Living condition*		
Living with immediate family	336	88.9
Living with extended family	12	3.2
Living alone	30	7.9

*Job or occupation*		
Government employee	80	21.2
Nongovernment employee	24	6.3
Farmer	115	30.4
Housewife	117	31.0
Others^*∗∗∗*^	42	11.1

*Ethnicity*		
Oromo	210	55.6
Amhara	112	29.6
Gamo	44	11.6
Others^*∗*^	12	3.2

*Religion*		
Muslim	165	43.6
Orthodox	153	40.5
Protestant	49	13.0
Others^*∗∗*^	11	2.9

*Level of income*		
<1000 EB	21	5.6
1000–2000 EB	89	23.5
2001–3000 EB	81	21.4
>3000 EB	187	49.5

*Residence*		
Rural	170	45.0
Urban	208	55.0

^*∗*^Accounts for the Welayta and Tigre ethnic group, ^*∗∗*^accounts for the Waaqeffata and Adventists religion group, ^*∗∗∗*^daily laborers and drivers, and EB = Ethiopian Birr.

**Table 2:** Baseline clinical characteristics of study participants at Bale Zone selected public hospitals, Oromia Region, Ethiopia, 2020 (*n* = 378).

Variables	Frequency	Percentage
*Family history of hypertension*		
Yes	202	53.4
No	176	46.6

*Family history of diabetes*		
Yes	183	48.4
No	195	51.6

*Time since diabetes diagnosis (years)*		
<5	198	52.4
5–10	116	30.7
>10	64	16.9

*Time since hypertension diagnosis (years)*		
<5	197	52.1
5–10	106	28.1
>10	75	19.8

*Frequency of follow-up*		
Monthly	266	70.4
Every two months	108	28.6
Every three months	4	1.1

*Blood glucose*		
Controlled	128	33.9
Uncontrolled	250	66.1

*BMI*		
Normal	182	48.1
Overweight	176	46.6
Obese	20	5.3

*Comorbidity*		
Asthma	35	9.3
Cardiovascular disease	61	16.1
Chronic kidney disease	50	13.2
Others^*∗*^	14	3.7
Do not have	218	57.7

*Stressed*		
Yes	40	10.6
No	338	89.4

*Adherence to an antihypertensive drug*		
Nonadherent	326	86.2
Adherent	52	13.8

^*∗*^Tuberculosis.

**Table 3:** Dietary and behavioral characteristics of study participants at Bale Zone selected public hospitals, Southeast Ethiopia, 2020/2021 (*n* = 378).

Variables	Frequency	Percentage
*Current alcohol drinker*		
Yes	122	32.3
No	256	67.7

*Frequency of alcohol consumption*		
Daily	6	1.5
5–6 days per week	12	3.2
1–4 days per week	28	7.4
1–3 days per week	38	10.1
Less than once per week	38	10.1
Do not use	256	67.7

*Do you smoke a cigarette?*		
Yes	61	16.1
No	317	83.9

*Do you smoke cigarettes now?*		
Current smoker	59	15.6
Ex-smoker	2	0.5
Never smoke	317	83.9

*Vigorous intensive activity*		
Yes	43	11.4
No	335	88.6

*Moderate intensive activity*		
Yes	127	33.6
No	251	66.4

*Do you walk or bicycle?*		
Yes	42	11.1
No	336	88.9

*Transportation*		
Use vehicle	352	93.1
On foot	23	6.1
Alternatives	3	0.8

*Eating animal sources of fatty flesh meat*		
Yes	272	72.0
No	106	28.0

*Eating high-calorie eggs*		
Yes	337	89.2
No	41	10.8

*Eating high-calorie food (burger, kukisi, and pizza)*		
Yes	51	13.5
No	327	86.5

*Salt intake level/day*		
Less than one teaspoon/day	208	55.0
One teaspoon/day	42	11.2
Above one teaspoon/day	22	5.8
Do not use/day	106	28.0

**Table 4:** Utilization of antihypertensive and hypoglycemic agents among study participants at Bale Zone selected public hospitals, 2020 (*n* = 378).

Medications	Frequency	Percentage
*Monotherapy (antihypertensive drug)*		
Nifedipine	22	5.8
Amlodipine	47	12.4
Hydrochlorothiazide	6	1.6
Atenolol	5	1.3
Enalapril	40	10.6

*Dual therapy (antihypertensive drug)*		
Enalapril + amlodipine	113	29.9
Hydrochlorothiazide + enalapril	73	19.3
Enalapril + atenolol	32	8.5
Hydrochlorothiazide + atenolol	12	3.2

*Triple therapy (antihypertensive drug)*		
Enalapril + amlodipine + hydrochlorothiazide	18	4.8
Furosemide + enalapril + amlodipine	10	2.7

*Antidiabetic agents (drugs)*		
Oral hypoglycemic agent	213	56.4
Insulin	141	37.2
Insulin + oral hypoglycemic agent	24	6.4

**Table 5:** Factors associated with uncontrolled hypertension among hypertension comorbid diabetic patients in Bale Zone public hospitals, Southeast Ethiopia, 2020/2021 (*n* = 378).

Variables	Level of hypertension		COR (95% CI)	AOR (95% CI)
	Controlled (%)	Uncontrolled (%)		
*Age*				
<50	40 (60.6)	147 (47.1)	1	1
≥50	26 (39.4)	165 (52.9)	1.72 (1.01, 2.97)^*∗*^	1.25 (0.60, 2.60)

*Sex*				
Male	38 (57.6)	173 (55.4)	1	1
Female	28 (42.4)	139 (44.6)	1.09 (0.64, 1.86)	0.82 (0.38, 1.72)

*Level of education*				
No formal education	18 (27.3)	84 (26.9)	1.31 (0.61, 2.81)	1
Primary education	22 (33.3)	103 (33)	1.10 (0.51, 2.41)	2.36 (0.96, 5.79)
Secondary education	17 (25.8)	78 (25)	0.84 (0.38, 1.84)	3.23 (1.18, 8.87)^*∗*^
Tertiary and above	9 (13.6)	47 (15.1)	1	3.82 (0.99, 14.73)

*Occupation*				
Government employee	19 (28.8)	61 (19.6)	1	1
Nongovernment employee	8 (12.1)	16 (5.1)	0.62 (0.231, 1.681)	0.55 (0.18, 1.67)
Farmers	19 (28.8)	106 (34)	1.93 (0.92, 4.03)	1.65 (0.65, 4.17)
Housewife	13 (19.7)	104 (33.3)	2.49 (1.15, 5.39)^*∗*^	2.56 (0.94, 6.9)
Others^*∗∗*^	7 (10.6)	25 (8)	0.99 (0.42, 2.39)	1.25 (0.38, 4.06)

*Currently smoke cigarette*				
Yes	15 (22.7)	46 (14.7)	0.59 (0.31, 1.13)	1.01 (0.41, 2.52)
No	51 (77.3)	266 (85.3)	1	1

*Excess alcohol drinker*				
Yes	28 (42.4)	94 (30.1)	0.59 (0.34, 1.01)	0.77 (0.35, 1.66)
No	38 (57.6)	218 (69.9)	1	1

*Do vigorous physical activity?*				
Yes	11 (16.7)	32 (10.3)	1	1
No	55 (83.3)	280 (89.7)	1.75 (0.83, 3.68)	0.74 (0.31, 1.76)

*Body mass index*				
18.5–24.99	20 (30.3)	141 (45.2)	1	1
25–29.99	41 (62.1)	146 (46.8)	0.51 (0.28, 0.90)^*∗*^	0.49 (0.211, 0.93)
≥30	5 (7.6)	25 (8)	0.071 (0.24, 2.06)	4.08 (1.24, 13.45)^*∗*^

*Time since diabetes diagnosed*				
<5 years	32 (48.5)	104 (33.3)	1	1
5–10 years	29 (43.9)	140 (44.9)	1.48 (0.85, 2.61)	1.26 (0.66, 2.42)
>10 years	5 (7.6)	68 (21.8)	4.18 (1.55, 11.27)^*∗*^	2.57 (0.80, 8.25)

*Time since HTN diagnosed*				
<5 years	38 (57.6)	115 (36.9)	1	1
5–10 years	25 (37.6)	125 (40.1)	1.65 (0.93, 2.90)	1.47 (0.66, 2.82)
>10 years	3 (4.5)	72 (23)	7.93 (2.36, 26.64)^*∗*^	5.2 (1.27, 21.38)^*∗*^

*Adherence to an antihypertensive drug*				
Nonadherent	48 (12.7)	278 (73.6)	3.06 (1.60, 5.86)^*∗*^	2.45 (1.10, 5.39)^*∗*^
Adherent	18 (4.8)	34 (8.9)	1	1

*Comorbidity*				
No	46 (69.7)	172 (55.1)	1	1
Yes	20 (30.3)	140 (44.9)	1.87 (1.058, 3.312)^*∗*^	1.71 (0.90, 3.26)

^*∗*^ *p* value less than 0.05, ^*∗∗*^daily laborers and drivers, COR: crude odds ratio, and AOR: adjusted odds ratio.

## Data Availability

The data used to support the findings of this study are available from the corresponding author upon reasonable request.

## Abbreviations

AOR: Adjusted odds ratio
ART: Antiretroviral therapy
BMI: Body mass index
COR: Crude odds ratio
DM: Diabetes mellitus
NCDs: Noncommunicable disease
SPSS: Statistical Package for Social Sciences.
